# How Shenzhen, China avoided widespread community transmission: a potential model for successful prevention and control of COVID-19

**DOI:** 10.1186/s40249-020-00714-2

**Published:** 2020-07-10

**Authors:** Huachun Zou, Yuelong Shu, Tiejian Feng

**Affiliations:** 1 School of Public Health (Shenzhen), Sun Yat-sen University, Shenzhen, China; 2 Shenzhen Center for Disease Control and Prevention, Shenzhen, China

**Keywords:** COVID-19, Outbreak, Control, Shenzhen, China

## Abstract

Shenzhen is a city of 22 million people in south China that serves as a financial and trade center for East Asia. The city has extensive ties to Hubei Province, the first reported epicenter of the coronavirus disease 2019 (COVID-19) outbreak in the world. Initial predictions suggested Shenzhen would experience a high number of COVID-19 cases. These predictions have not materialized. As of 31 March 2020 Shenzhen had only 451 confirmed cases of COVID-19. Contact tracing has shown that no cases were the result of community transmission within the city. While Shenzhen did not implement a citywide lockdown like Wuhan, it did put into place a rapid response system first developed after the severe acute respiratory syndrome (SARS) epidemic in 2003. In the wake of the 2003 SARS outbreak, Shenzhen health authority created a network for surveillance and responding to novel respiratory infections, including pneumonia of unknown causes (PUC). The network rapidly detected mass discussion about PUC and immediately deployed emergency preparedness, quarantine for close contacts of PUC. Five early actions (early detection, early reporting, early diagnosis, early isolation, and early treatment) and four centralized responses (centralized coordination by experts, centralized allocation of resources, centralized placement of patients, and centralized provision of treatment) ensured effective prevention and control. Tripartite working teams comprising community cadres, medical personnel and police were formulated to conduct contact tracing at each neighborhood and residential community. Incorporation of mobile technology, big data, and artificial intelligence into COVID-19 response increased accessibility to health services, reduced misinformation and minimized the impact of fake news. Shenzhen’s unique experience in successfully controlling the COVID-19 outbreak may be a useful model for countries and regions currently experiencing rapid spread of the virus.

## Background

Shenzhen is a city of 22 million people in south China that serves as a financial and trade center for East Asia. The city has extensive ties to Hubei Province, the first reported epicenter of the coronavirus disease 2019 (COVID-19) outbreak in the world. Shenzhen Center for Disease Control and Prevention (CDC) estimated over 2 million of Shenzhen’s permanent residents are migrants from Hubei Province, and over 1 million people traveled between Shenzhen and Hubei Province in the four-week period prior to the Wuhan lockdown on 23 January 2020. Due to the interconnectedness of the two regions and high basic reproduction rate (*R*_0_) [1], initial predictions suggested Shenzhen would experience a high number of COVID-19 cases. These predictions have not materialized.

**Fig. 1:**
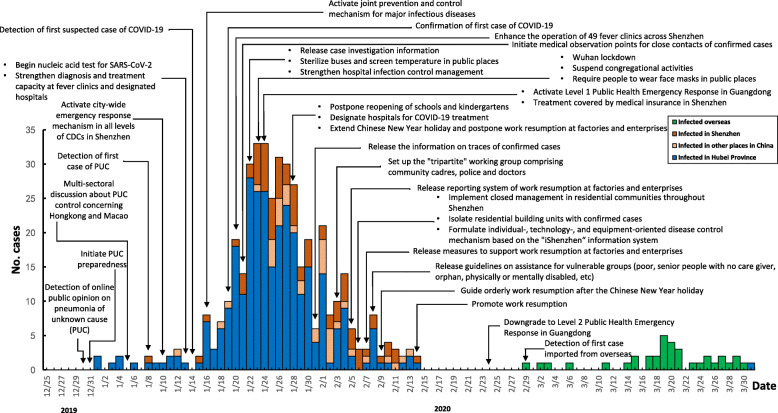
Response to COVID-19 in Shenzhen, China

As of 31 March 2020 Shenzhen had only 451 confirmed cases of COVID-19. Of these, 376 (83%) were infected outside of Shenzhen -- 304 in Hubei, 39 in other parts of China, and 33 in other countries. Only 75 cases were infected in Shenzhen, and contact tracing has shown that no cases were the result of community transmission within the city [2]. While Shenzhen did not implement a citywide lockdown like Wuhan, it did put into place a rapid response system first developed after the severe acute respiratory syndrome (SARS) epidemic in 2003. Better coordination, cooperation, and strong solidarity is essential in the joint efforts of fighting against COVID-19 [3]. There has been a rapid surge in research in response to the outbreak of COVID-19 [4]. Shenzhen’s unique experience in successfully controlling the COVID-19 outbreak may be a useful model for countries and regions currently experiencing rapid spread of the virus (Fig. 1).

### Early action and centralized responses

In the wake of the 2003 SARS outbreak, Shenzhen health authority created a network for detecting and responding to novel respiratory infections, including pneumonia of unknown cause (PUC). The pillars of this system include five early actions (early detection, early reporting, early diagnosis, early isolation, and early treatment) and four centralized responses (centralized coordination by experts, centralized allocation of resources, centralized placement of patients, and centralized provision of treatment). On 30 December 2019 this network detected widespread online discussions about PUC in Wuhan. The following day centralized response mechanisms were activated. The Shenzhen CDC organized an emergency team of public health leadership, epidemiologists, lab scientists, and clinicians to oversee and direct the response to a possible emerging outbreak. Within 5 days the team had designated a single hospital as the centralized treatment site for all PUC in Shenzhen and coordinated with local customs authorities to establish protocols for screening and isolating all travelers from Wuhan. Once SARS-CoV2 had been identified as the cause of these cases of PUC, the Shenzhen CDC laboratory coordinated with national healthcare authorities to obtain approval to conduct confirmatory testing locally and directed the city’s 39 influenza surveillance network laboratories to convert their existing infrastructure to test for SARS-CoV2. The world did not know whether COVID-19 virus can cause human-to-human transmission at its early stage. The PUC system in Shenzhen detected the first SARS-CoV2 case in Guangdong province and found one of the first human-to-human transmission cases in China. A female who had never been to Hubei but exposed to other six family members (four confirmed COVID-19) returning from Hubei was confirmed with COVID-19 on 14 January 2020 [5]. Based on this cluster and other evidence, on 20 January 2020, the high-level expert panel of the National Health Commission led by Dr. Nanshan Zhong warned the potential human-to-human transmission.

With early diagnosis and isolation measures in place, the team turned to address public concerns about the virus. On 24 January a COVID-19 hotline was set up for the community to seek help, and Shenzhen city government announced that COVID-19 treatment would be fully covered under public healthcare insurance. As the number of confirmed COVID-19 cases grew in Shenzhen, the COVID-19 Prevention and Control Command Station (CPCCS) was activated on 28 January to coordinate expanded outbreak control measures throughout the city. Ten working groups were formed (epidemic monitoring, laboratory and diagnostics, sanitization, logistics, information dissemination, medical observation, risk assessment, biosafety management, scientific research, and command and control), and 720 public health personnel were mobilized to conduct case finding and contact tracing for each new confirmed case of COVID-19.

### Neighborhood outbreak teams

More than 660 multisectoral teams of community cadres, medical personnel, and police officers were organized to carry out prevention and control measures at the neighborhood level. In each residential community, cadres disseminated information to residents and controlled movement into and out of public spaces, with support from police officers. Medical personnel provided on-site health assessments, psychological support, COVID-19 testing, case reporting, and referrals to treatment. Community social workers delivered of food and medicine to residents sheltering at home. Teams shared data with CPCCS working groups via WeChat (the most popular social app in China) on a daily basis.

### Leveraging technology

As a global hub for information technology (IT) and home to leading analytics companies, Shenzhen was able to incorporate mobile technology, big data, and artificial intelligence into its COVID-19 response. Starting 31 January 2020 de-identified information for every confirmed and suspected case of COVID-19 was released on the Shenzhen Municipal Government website [6]. Publicly available information included gender, age, activity route, date of diagnosis, and number of close contacts. Local IT companies soon incorporated this information into online maps that residents could reference to track cases in their vicinity and make informed decisions about social distancing and other preventative measures.

### Caring for vulnerable persons

Certain groups are at increased risk of being negatively impacted by COVID-19 prevention and control measures, including seniors without caregivers, orphans, people living with physical or mental disabilities, the poor, and homeless persons. Municipal funds were allocated to support these groups. For all vulnerable persons who were either confirmed or suspected cases of COVID-19, city government agreed to provide a subsidy of up to 24 months of minimum salary as well as clothing, food, drinking water, face masks, sanitizing materials, and psychological support [7].

### Difficulty or challenges

Difficulty or challenges existed in the implementation of interventions. For example, some premises did not strictly abide by body temperature monitoring protocols; some individuals did not fully cooperate with quarantine guidelines; some people intentionally hid their COVID-19 status, which made it very hard to do contact tracing of their close contacts; some factories and companies did not duly apply social distancing measures after work resumption. The government issued guidelines on COVID-19 containment measures and clearly outlined punishment for infringement of these protocols.

### Future challenges

As travel bans are lifted and life begins to return to normal across China, millions of workers from Hubei Province are expected to return to Shenzhen. The need for intensive case finding and contact tracing is likely to continue for months, if not years. Factories, which employ much of Shenzhen’s migrant population, will be a particularly challenging environment in which to implement regular screening and testing. Additionally, Shenzhen borders Hong Kong, and the return of international travellers threatens to bring a new wave of imported COVID-19 cases requiring additional prevention and control measures.

## Conclusions

As community transmission of COVID-19 accelerates abroad, Shenzhen has recorded no local infections since 22 February 2020. By implementing a rapid response system that included five early actions and four centralized responses, Shenzhen was able to quickly recognize an emerging epidemic and control spread of the disease. Multisectoral coordination, proactive contact tracing and testing, timely isolation and treatment, hospital infection control, effective community management, and prompt information dissemination were key actions toward containing the epidemic and reassuring citizens. Similar responses may be implemented in countries and regions that are preparing responses to the COVID-19 pandemic.

## Data Availability

Not applicable.
